# Measuring and Characterizing the Human Nasal Cycle

**DOI:** 10.1371/journal.pone.0162918

**Published:** 2016-10-06

**Authors:** Roni Kahana-Zweig, Maya Geva-Sagiv, Aharon Weissbrod, Lavi Secundo, Nachum Soroker, Noam Sobel

**Affiliations:** 1 Department of Neurobiology, The Weizmann Institute of Science, Rehovot, 76100, Israel; 2 Loewenstein Rehabilitation Hospital, Ra’anana, 43100, Israel; 3 Sackler Faculty of Medicine, Tel Aviv University, Ramat Aviv, 69978, Israel; Francis Crick Institute, UNITED KINGDOM

## Abstract

Nasal airflow is greater in one nostril than in the other because of transient asymmetric nasal passage obstruction by erectile tissue. The extent of obstruction alternates across nostrils with periodicity referred to as the nasal cycle. The nasal cycle is related to autonomic arousal and is indicative of asymmetry in brain function. Moreover, alterations in nasal cycle periodicity have been linked to various diseases. There is therefore need for a tool allowing continuous accurate measurement and recording of airflow in each nostril separately. Here we provide detailed instructions for constructing such a tool at minimal cost and effort. We demonstrate application of the tool in 33 right-handed healthy subjects, and derive several statistical measures for nasal cycle characterization. Using these measures applied to 24-hour recordings we observed that: 1: subjects spent slightly longer in left over right nostril dominance (left = 2.63 ± 0.89 hours, right = 2.17 ± 0.89 hours, t(32) = 2.07, p < 0.05), 2: cycle duration was shorter in wake than in sleep (wake = 2.02 ± 1.7 hours, sleep = 4.5 ± 1.7 hours, (t(30) = 5.73, p < 0.0001). 3: slower breathing was associated with a more powerful cycle (the extent of difference across nostrils) (r = 0.4, p < 0.0001), and 4: the cycle was influenced by body posture such that lying on one side was associated with greater flow in the contralateral nostril (p < 0.002). Finally, we provide evidence for an airflow cycle in each nostril alone. These results provide characterization of an easily obtained measure that may have diagnostic implications for neurological disease and cognitive state.

## Introduction

Cyclic events constitute a fundamental aspect of biological function at levels ranging from sub-cellular components to the entire organism [1]. One such large-scale cycle evident in mammals is known as the nasal cycle, where nasal airflow is greater in one nostril than in the other, and the greater airflow nostril shifts between left and right over time [2]. The nasal cycle was apparent in all mammalian species where it was investigated [3–10].

The physical mechanism underlying the nasal cycle is an asymmetry in blood flow leading to engorgement of erectile tissue in the anterior part of the nasal septum and inferior turbinate of one nostril over the other [11]. This asymmetrically enlarged tissue physically blocks the passage of air in one nostril more than in the other. Although this physical mechanism has been identified, the physiological mechanisms that drive it are not equally well understood. These mechanisms are clearly related to the autonomic nervous system in that unilateral sympathetic dominance is associated with vasoconstriction and decongestion in one nostril, while simultaneous parasympathetic dominance is associated with vasodilatation and congestion in the other [12–14]. That said, autonomic nervous system asymmetry may not be the only driver of the nasal cycle given that a cycle is observed after thoracotomy, vagotomy and vidian neurectomy [9] and in patients with autonomic nervous disturbance [15]. In addition, there was no correlation between the nasal cycle and facial temperature [16] or middle ear pressure [17] that one would expect under asymmetric autonomic system modulation alone.

The functional role of the nasal cycle is debated. Some studies explain the alternation in nasal airflow as a mechanism for air conditioning and the removal of entrapped contaminants [18], or for mucociliary clearance [19]. Others hypothesize that the nasal cycle is involved in protection against respiratory infection or allergies or indicates these physiological states [20–23]. These theories refer to the nose as a respiratory organ and largely ignore olfaction and the nasal cycle–CNS relation. We have proposed an olfaction-centered theory for the functional significance of the nasal asymmetry in airflow. Specifically, a given nasal airflow optimizes perception of a given set of odorants as a function of odorant solubility [24]. Because the nasal cycle results in a different airflow in each nostril, it optimizes each nostril for different odors. This culminates in two offset olfactory images, one from each nostril, which are simultaneously sent to the brain with each sniff [25]. Together, this provides for a greater overall olfactory range [26]. Although this theory implies a functional sensory consequence for nasal flow asymmetry, it does not provide an explanation for why this asymmetry should cycle. That said, one might raise the possibility that cycling would then optimize olfactory perception for different types of odors at different physiological states, e.g., food optimized versus predator optimized.

The nasal cycle can change with body posture [22,27–30] changes with age [31–37] is related to handedness [38] and is also reflected in a host of non-olfactory brain activity measures such as electroencephalography (EEG) and cognitive-task performance. The laterality (ipsi vs. contra) of this relation, however, remains unclear, with different studies finding increased brain activity either ipsilaterally [39] or contralaterally to the high airflow nostril as evidenced in EEG [40–42] and behavior [43–47]. Moreover, to what extent functional brain asymmetry drives nasal airflow asymmetry or nasal airflow asymmetry drives functional brain asymmetry remains unresolved [48–52]. Nevertheless, obstructing one nostril and forcing uni-nostril respiration can alter various tasks and performance [27,39,41,43–46,49,53–55] as well as EEG parameters [48,50]. The nasal cycle is also related to various physiological measures beyond direct brain measurement such as heart rate and blood pressure [55,56], glucose blood levels [57], intraocular pressure [58–60] blink rate [61], and alternating lateralization of plasma catecholamines [62]. Finally, the nasal cycle is altered in a host of neurological and non-neurological conditions including high spinal cord injuries [63], autism [64], Parkinson’s disease [65], schizophrenia [66] Kallmann's syndrome [67], cardiac symptoms [68], fever and electrolyte imbalance [69]. Thus, characterization of the nasal cycle may have significant diagnostic value for neurological conditions and beyond.

Previous studies in humans found that the nasal cycle periodicity, i.e., the shift in greater airflow or lower resistance from one nostril to the other, ranges from 25 min to 8 h with peak interval between 1.5–4 h during wake [13,70,71]. These values were obtained with methods for nasal cycle measurement that have evolved with technology. Temporally discrete measurements can be made by simply exhaling through the nose onto a mirror [72,73] or by applying various advanced measures of acute nasal flow such as rhinoresistometry, rhinomanometry, acoustic rhinometry and flexible liquid crystal thermography [32,35,74–79]. The nasal cycle has also been observed using discrete MRI structural imaging of the turbinates [80–82]. The advantage of the temporally discrete airflow methods is in their accuracy, but when used to characterize an ongoing cycle one needs to have subjects return for measurement at high frequency, and moreover, such discrete measurements cannot be applied in sleep. In turn, continuous measurements can be made using thermal [75] and auditory sensors [83], or using small pressure sensors at the far end of tubes nestled at the nasal opening. Whereas several such efforts have been extended [84–86], a commercial device for assessing the nasal cycle over time remains an expensive option in a large-scale medical device (e.g., http://www.orl.biofonia.com/rcs_gene/medical_equipment/Leaflet_RHINO-SYS_GAESaudiotest_ENG.pdf), and the aforementioned studies did not provide details that would allow the interested scientist to build their own simple tool.

The above reviewed literature implies that the nasal cycle is a potential indicator of autonomic arousal in health and disease. With this in mind, the modest goals of this manuscript are two: First, to generate detailed instructions that will allow construction of an accurate and robust nasal cycle logger with minimal effort and cost, combined with detailed instructions for statistical derivations of nasal cycle periodicity. The second goal of this manuscript is to provide added characterization of this phenomenon in a relatively large cohort.

## Methods

### Building a nasal airflow logger

**Fig 1:**
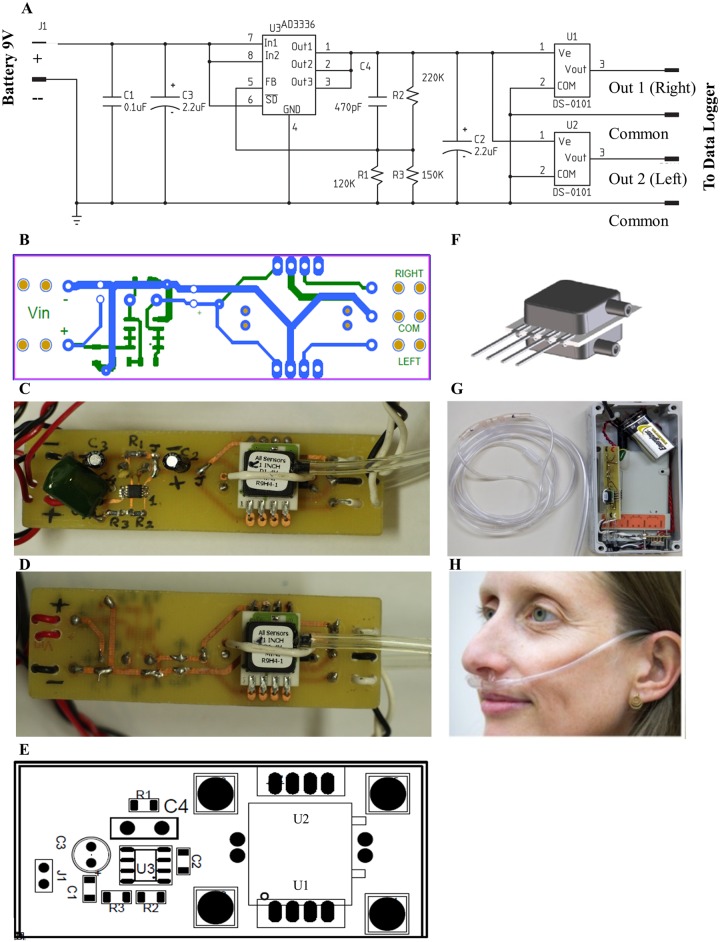
Schematic for nasal cycle logger. (A) Schematic of electronics. (B) Printed circuit board (PCB). Component-side in green, soldering-side in blue. (C) Picture of component-side. (D) Picture of soldering-side. (E) Component layout. (F) Illustration of low Pressure Sensor (1” H_2_O to 30”H_2_O). (G) The device in its assembled form. (H) Respiration cannulas positioned in subject’s nares.

There is an extensively established powerful linear relation between nasal airflow velocity and nasal pressure [87]. With this relation in mind we use the terms nasal pressure and nasal airflow interchangeably in this manuscript. By placing a pressure sensor at the far end of a nasal cannula one can measure nasal airflow velocity with such sensitivity that it discriminates the flow in response to one odor versus another [88]. Here we use a readily available cannula (REF 4804*, Demand Nasal Canula (Adult), Salter Labs) with separated tubes for each nostril (a cannula with a septum) connected to a small wearable device. The device includes two high-sensitivity pressure sensors (AllSensors 1 INCH D1-4V MINI) connected to a data logger (ACR Smart Reader Plus 7, 1.5 Mb) providing for 26 hours of continuous recording at 5.5 Hz. The conversion ratio between inch H2O to volt using these sensors is 1 inch H2O = 2v. One inch H2O also equals 249.1 Pascal. Therefore 1V = 124.55 Pascal. Note that whereas we used a high-end logger that was available to us, several cheaper options with more than sufficient performance are available (e.g. http://www.inds.co.uk/test/msr145.htm). The full bill of materials for building an OEM device is in Table 1, the device electronics schematic is in Fig 1A–1E, and a photo of the device and a person wearing it is in Fig 1G and 1H. The total cost of the device is ~$500 and it takes a few hours to assemble.

**Table 1:** The full bill of materials for building a nasal cycle monitor.

Part description	Specific Part Model	Number of units needed	Possible Supplier	Estimated part’s Cost
Data Logger	MSR 145 (we used SmartReaderPlus)	1	MicroDAQ.com	$367
**Circuit board:**				
Printed circuit board	Fiberglas 77x23 mm board	1	Any electronics supply shop	~$1
Pressure sensors	1” H2O	2	All Sensors 1inch D1-4V Mini	$39.50 x 2 = $79
Dual port cannula	REF: 4804	1	Salter Labs	$2
Package box	364–8425	1	RS (can be any electronics supply shop)	$15
**Circuit board components**				
Resistor	120 Kohm	1	RS, Farnell, etc.	~ $1 / 100 pcs.
Resistor	150 Kohm	1	RS, Farnell, etc.	~ $1 / 100 pcs.
Resistor	220 Kohm	1	RS, Farnell, etc.	~ $1 / 100 pcs.
Capacitor	0.1 uF, ceramic	1	RS, Farnell, etc.	~ $1 / 100 pcs.
Capacitor	2.2 uF, 16 Volt	2	RS, Farnell, etc.	~ $1 / 100 pcs.
Capacitor	470 pf ceramic	1	RS, Farnell, etc.	~ $1 / 100 pcs.
5V voltage regulator	ADP3336	1	Analog Devices	$2.5

### Logger data pre-processing

**Fig 2:**
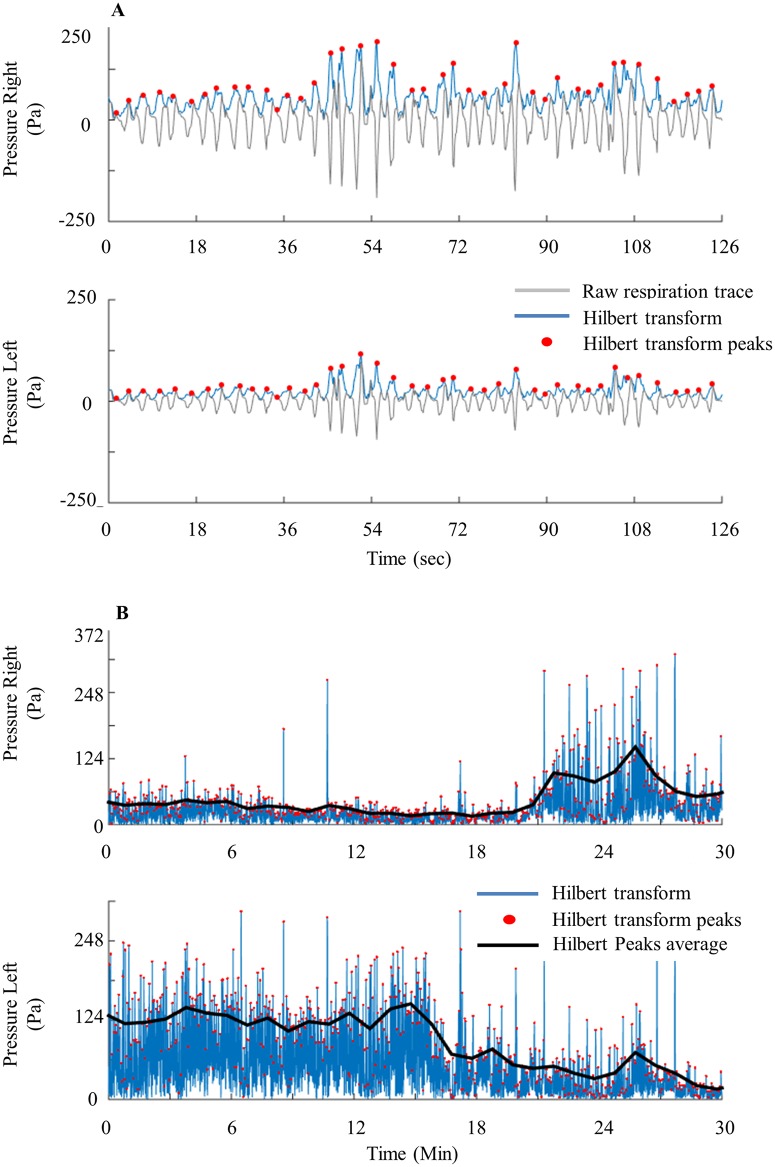
Pre-processing stages. (A) Raw data overlaid with Hilbert transform and its peaks during 2 minute time scales. (B) Hilbert transform overlaid with its peaks and average during 30 minute time scales.

After collecting the dual nasal airflow trace we followed several preprocessing stages: 1) Removal of DC offset by subtracting the mean of the respiratory trace. 2) Using a Hilbert-transform to extract the amplitude of each inhale-exhale cycle. 3) Assessment of airflow in each nostril by extracting the envelope of the inhale-exhale cycles. This can be done using the ‘*findpeaks*’ function in Matlab (Mathworks inc) on the Hilbert transformed respiratory trace, and then averaging the peaks over each non-overlapping one-minute time windows to reduce noise. The results of this preprocessing stage are two time-series representing the average airflow in each nostril. These two airflow time-series will be labeled as Flow_R_ and Flow_L_ for right and left nostril airflow respectively (Fig 2).

### Measure derivation

**Fig 3:**
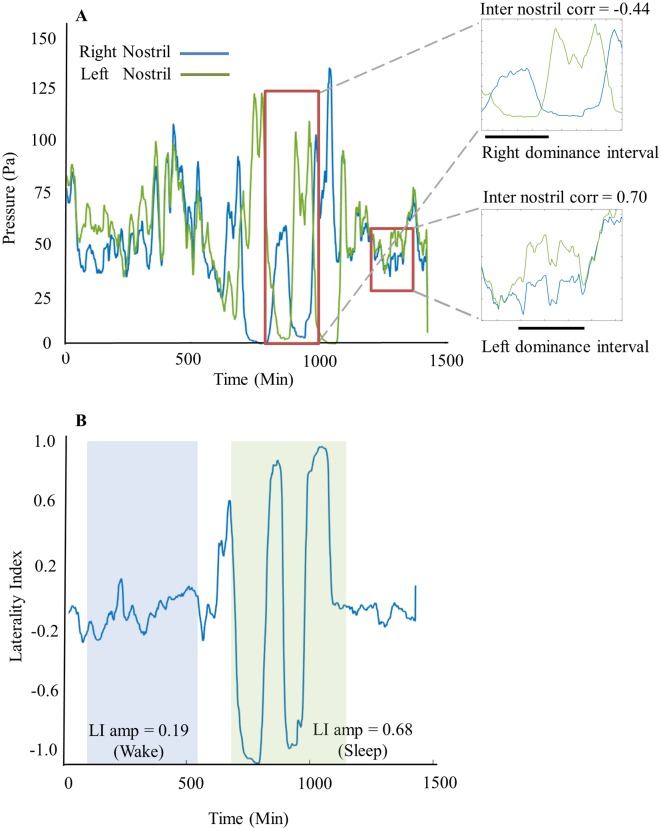
Example of processed data from a typical subject. (A) Average filtered nasal airflow peaks over time for right (blue) and left (green) nostrils (smoothed with a 20 minute window for display). Large red rectangle highlights a portion of sleep with right dominance indicated by black bar and negative inter nostril correlation (r = -0.44). Small red rectangle highlights a portion of wake with left dominance indicated by black bar and positive inter nostril correlation (r = 0.7). (B) Laterality index graph calculated and aligned for the data presented above. Light blue shading highlights low LI amplitude in wake (mean = 0.19) and light green highlights high amplitude in sleep (mean = 0.68).

A major goal of this study beyond instructions for constructing the recording device is the development of a set of statistical measures that can be used to characterize the nasal cycle. We set out to derive the following measures (Fig 3):

#### 1. Respiration Laterality Index (LI)

This index measures the flow ratio between the left and right nostril. LI is calculated using the following equation: $\text{LI}=\frac{\left(\text{Flo}{\text{w}}_{R}-\text{Flo}{\text{w}}_{L}\right)}{\left(\text{Flo}{\text{w}}_{R}+\text{Flo}{\text{w}}_{L}\right)}$ for every minute. The result of this calculation is a one minute resolution time series of the lateralization extent, with a value of 1 representing airflow only through the right nostril (Flow_*L*_ = 0), a value of -1 representing airflow only through the left nostril (Flow_*R*_ = 0), and a value of 0 representing equal flow through left and right nostrils (Flow_*R*_ = Flow_*L*_). Using the LI vector we could then derive:

**Cycle periodicity:** This measures the interval length (in minutes) at which the Respiration Laterality Index does not change sign, in other words this measures the length in minutes in which each nostril was dominant. The interval length is calculated by measuring the time difference between two consecutive zero crossings of the LI (i.e. the time difference from when the LI switches from negative to positive till when it switches from positive to negative or vice versa). Intervals shorter than 15 minutes were considered noise. The result of this calculation is a list of interval lengths for each subject. These intervals were assigned to left dominance intervals vs. right dominance intervals or wake intervals vs. sleep intervals.

**Mean LI:** The average of the LI vector over a chosen time period. The **mean LI** value represents whether a specific subject had a tendency to be in left or right nasal cycle dominance over a chosen time period.

**Mean LI amplitude:** The average of the absolute value of LI, over chosen time periods. The mean LI amplitude value represents to what extent a specific subject had nostril dominance (regardless of whether it was left or right); with a value of 1 representing high nostril dominance and a value of 0 representing equal flow through the nostrils.

#### 2. Inter-nostril correlation

This measures the correlation between the flows of the right and left nostrils. This is calculated by applying the Matlab *corrcoef* function to the Flow_R_ and Flow_L_ traces. This measure is important because it has been used extensively before [89,90] thus permitting comparison across studies. Moreover, it informs on whether there is one oscillator or two synchronized/unsynchronized oscillators underlying the nasal cycle.

#### 3. Nostril autocorrelation

Autocorrelation of each subject’s nostril’s airflow as well as each subject’s laterality index vector was fitted with a decaying cosine function, employing a method originally developed for characterizing oscillations in neuronal data [91,92]. Autocorrelation was calculated on the one-minute binned airflow data and was fitted with the following equation:

$$\text{y}(\text{t})=\text{a}\cdot {\text{e}}^{-\frac{\left|\text{t}\right|}{{\text{t}}_{\text{1}}}}\cdot (1+\text{cos}(2\pi \text{ft}))+\text{b}\cdot {\text{e}}^{-\frac{\left|\text{t}\right|}{{\text{t}}_{\text{2}}}}+\text{c}\cdot {\text{e}}^{-{\left(\frac{\text{t}}{{\text{t}}_{\text{3}}}\right)}^{2}}+\text{d}$$

where t is the autocorrelation time variable (from -1000 min to 1000 min for most subjects (depending on recording time). Fit was calculated for time points where the autocorrelation value was > 20% of autocorrelation peak whereby a, b, c, f, t1, t2, t3 are the fit parameters. Parameters were restricted to the following values: a = [0, m], b = [0, m], c = [–m, m], d = [0, m], f = [20, 500] 1/min, t_1_ = [5, 500] min, t_2_ = [0.1, 600] min, τ_3_ = [0, 5] min; where m is the maximum value of the autocorrelation. We employed a variation [89] of the original method [90]. In this variation the decay time-constant is separated into two separate time-constant parameters, one (t_1_) capturing the decay of the oscillatory component, and the other (t_2_) capturing the decay of the overall airflow. Importantly, this version includes a baseline component (d), which allows a good fit when using a long time window as we do in this study. Based on these parameters, one ‘best frequency’ is selected for each trace—usually the slow, higher-amplitude frequencies are better detected than fast shallow ones. To select the best fit, we performed the fitting 500 times with different random initial values for all parameters; re-computed the R^2^ value between the original autocorrelation and the fit for each solution, and chose the frequency based on the fit with the highest R^2^ value. To assess the significance of the oscillatory component (f) we required three criteria [91]: (1) We computed the 95% confidence interval of the oscillation-amplitude component (parameter ‘a’), and required that the fitted parameter a should be significant–namely, that a should fall outside the 95% confidence-interval. (2) The oscillation-amplitude component had to be ≥ 0.05, signifying modulation depth of at least 5% of the total amplitude of the airflow. (3) Complete decay of the oscillatory component (3×t_1_) should last for at least one period of the oscillation (1/f). In our analysis we included fluctuation frequencies smaller than 400 minutes due to the data sampling length (24 hours = 1440 minutes enabling at least 3 occurrences of the rhythm).

### Relation between body posture and nasal dominance

The nasal cycle is influenced by body posture. During dorsal recumbence nasal resistance increases on the more congested side, and in lateral recumbence resistance increases in the lower nasal cavity [27]. This relation reflects a reflex change in nasal vasomotor activity [22]. With progressively longer periods of lateral recumbence the nasal response increases in magnitude, endures for longer, producing a sustained phase reversal [28,29,93]. In order to investigate the relation between body posture and respiration each subject wore, in addition to the respiration logger, a miniature three axes acceleration data logger (HOBO Pendant G Data Logger, UA-004-64, Onset HOBO data loggers). The logger recorded body movements in x-y-z axes at 0.16 Hz. Position data was synchronized to the respiration meter. Each subject was instructed to carry the acceleration data logger on his/her left side of waist and to indicate in a log whether this position was changed during recording time. Position data was later assigned to 9 states: standing + 8 supine positions, each covering 45° as follows: On stomach, right/stomach, right, right/back, back, back/left, left, left/stomach.

### Subjects

In a validation test, we studied 33 healthy subjects (18F, mean age = 30.3 ± 9.9 years). Subjects were screened for right hand dominance using the Edinburgh Handedness Inventory—Short Form [94], and no history of nasal insults or respiratory diseases. All subjects provided written informed consented to procedures approved by the Loewenstein Rehabilitation Hospital Helsinki Committee.

### Procedures

Each subject was fitted with the device on the morning of the experimental day and was instructed to return to lab on the following morning at the same time. Subjects were provided with a diary in which they were requested to briefly describe their activity every 30 minutes during wake, and note time of going in and out of bed for nighttime sleep.

### Raw data availability

All the raw data collected in this study are available for download at: 10.6084/m9.figshare.3807564.

## Results

### The device effectively measured dynamic asymmetry of nasal congestion

**Fig 4:**
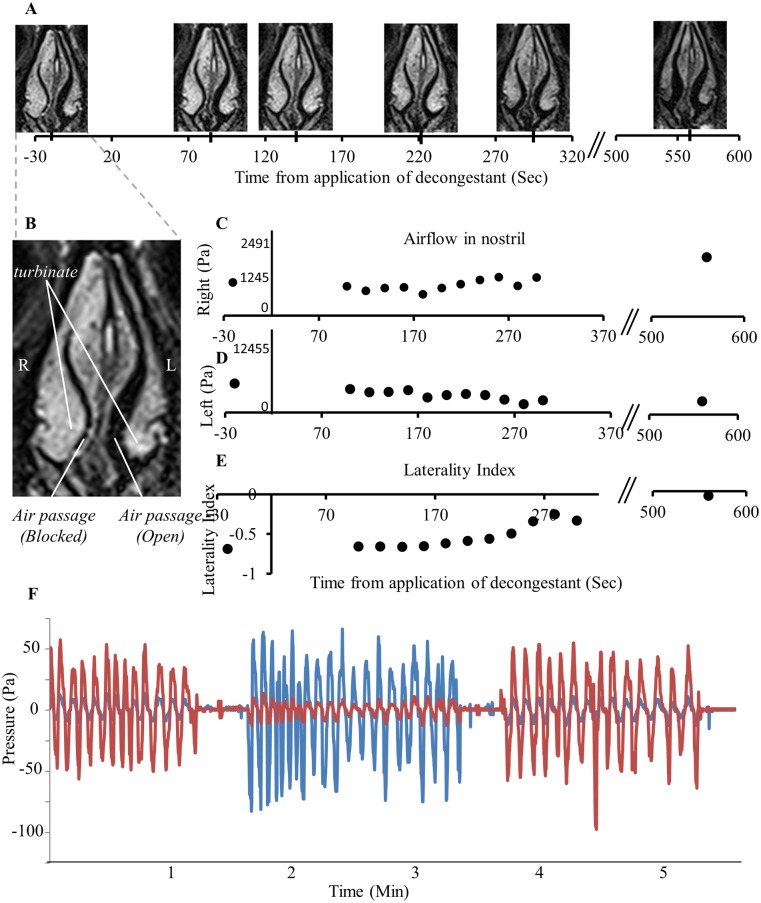
The device data reflected nasal obstruction. (A) Time Series of MRI scans showing the nasal turbinates. Time measured from application of Otrivin (0.1% Xylometazoline Hcl) to the right nostril. Note right nostril decongestion over time. (B) Larger image of nasal passage. (C) Airflow measured in right nostril. (D) Airflow measured in left nostril. (E) Laterality index calculated as: $\text{LI}=\frac{\left(\text{Flo}{\text{w}}_{R}-\text{Flo}{\text{w}}_{L}\right)}{\left(\text{Flo}{\text{w}}_{R}+\text{Flo}{\text{w}}_{L}\right)}$. (F) Airflow in one subject with flipping of left and right nasal cannulas every 1.5 minutes. One logger channel in red and one in blue.

As noted in the introduction, alterations in airflow follow asymmetric swelling of erectile tissue in the nostrils. To validate that this is indeed what we are measuring, we applied the measurement device to a subject directly after applying a nasal decongestant to one nostril (0.1% Xylometazoline Hcl, brand name: Otrivin), and concurrently obtained nasal airflow measurements and structural magnetic resonance images (MRI) of the nasal passage for 10 minutes. Consistent with our working hypothesis, we observed that as unilateral nasal swelling decreased, unilateral nasal airflow increased, and the nasal cycle shifted accordingly (r = 0.83, p < 0.001, Fig 4). In other words, the device measures the intended process. Finally, one may raise the concern that the measurement device itself, or that idiosyncrasy in its application, introduced artifactual asymmetries in recording. Such variance can reflect both within subject events such as abrupt motion or physical obstruction of a naris, or across subjects variation following different placement of the nasal cannula. To address within subject variation we observed that abrupt changes in body posture as measured by the position logger were not associated with abrupt changes in nasal cycle (likely reflecting our 15 minute filter). This data is detailed later in the section on the relation between body posture and nasal cycle. To address impact of across subject variation as well as potential device asymmetry we conducted the following experiment: We fitted the device to 10 users, measured for 1.5 minutes, then flipped left and right channels before measuring an added 1.5 minutes, and then flipped back to measure a final 1.5 minutes. The three measurements were highly correlated (all r > 0.89, all p < 0.001), and critically, subtracting the laterality index across measurement epochs generated an offset not significantly different from zero (all t(9) < 1.3 all p > 0.22) (Fig 4F). We conclude that idiosyncrasy in application and associated motion may indeed slightly alter absolute values, but they do not significantly impact nasal cycle determination.

### Subjects spent slightly longer in left over right nostril dominance

**Fig 5:**
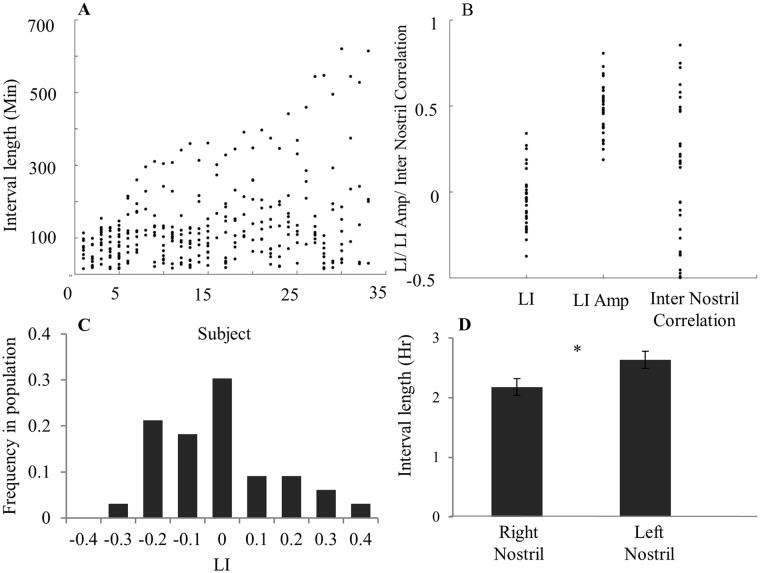
The nasal cycle was highly variable within and across subjects. (A) All intervals across all subjects. X-axis describes each subject (sorted by increasing variability), Y-axis describes all intervals measured for each subject during 24 hours. Each dot is an interval. (B) Range in parameters across subjects. Each dot is a subject. (C) LI distribution across the population. (D) Mean right vs left dominance intervals during 24 hours, reflecting a small but significant tendency to spend more time in left dominant intervals than in right dominant intervals. Error bars are SE.

**Fig 6:**
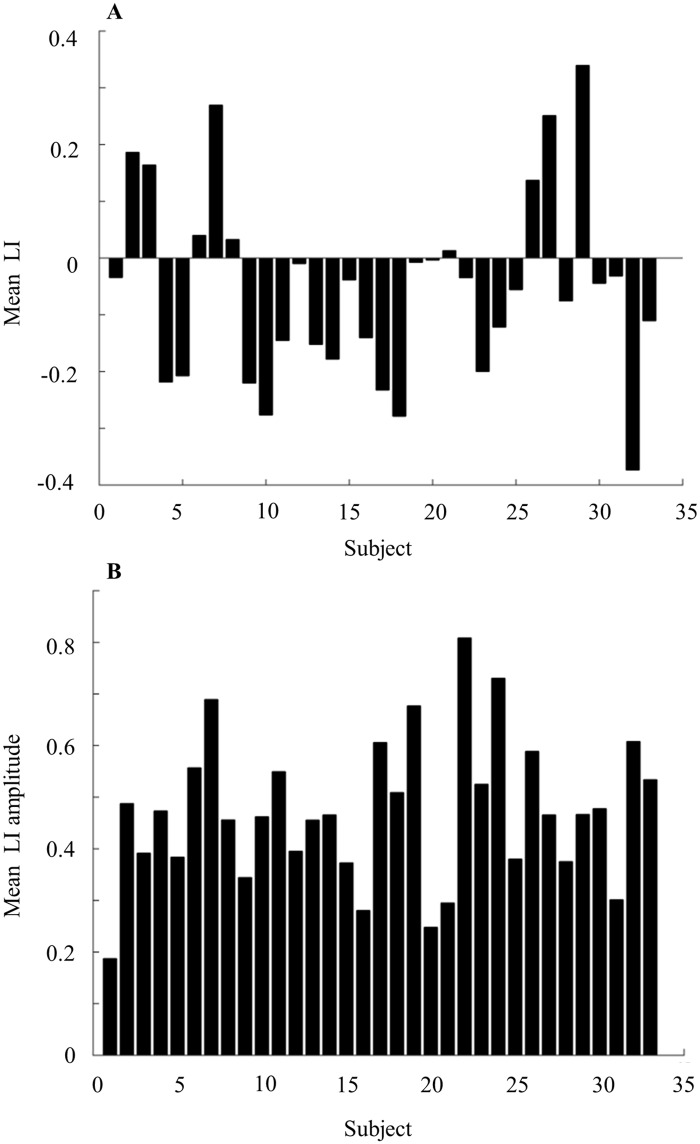
Laterality index measures for all subjects. (A) Mean laterality index over 24 hours for 33 all subjects. (B) Mean laterality index amplitude over 24 hours for all 33 subjects.

There is some variance in the literature as to what constitutes a "cycle" [36,90,95]. Here we define the presence of a cycle as at least one occurrence of nostril dominance change. Diurnal patterns of 33 healthy subjects (18F, mean age = 30.3 ± 9.9 years) exhibited large variability in nasal cycle length across subjects (Fig 5A). Although all subjects cycled, cycle length, i.e. the time interval of one nostril dominance, ranged from 15 min (minimum allowed by analysis) to 10.35 hours. The population average cycle length was 2.15 ± 1.84 hours. The population average mean LI over 24 hours was close to 0 (-0.05 ± 0.17), implying balanced dominance between right and left nostrils over time (Fig 5B). However, a scatter plot of all subject's mean-LIs uncovers the variability across subjects, with cases ranging in mean-LI between -0.4 and 0.4 (Fig 5B). This implies that during the recorded period many individuals had an asymmetric nostril-dominance, with one nostril less occluded than the other for a large proportion of the 24-hour recording (e.g. subjects 29 and 32 in Fig 6A). Across the population, mean right nostril interval was 2.17 ± 0.89 hours and mean left nostril interval was 2.63 ± 0.89 hours (paired t-test, t(32) = 2.07, p < 0.05, Fig 5D). In other words, consistent with recent findings [96], this population of all right-handed individuals spent slightly more time in left-nostril dominance. Mean LI amplitude over 24 hours ranged from 0.2 to 0.8 (mean = 0.47 ± 0.14, Figs 5B and 6B). This implies that uni-nostril dominance occurs during a large portion of the 24-hour cycle. Finally the inter nostril correlation ranged from -0.5 to 0.8 (mean = 0.12 ± 0.4, Fig 5B). This implies that the nasal cycle phenomenon is not limited to reciprocal nostril congestions’ and can also occur while nostril congestions’ are conjugated.

### Cycle duration was shorter in wake than in sleep

**Fig 7:**
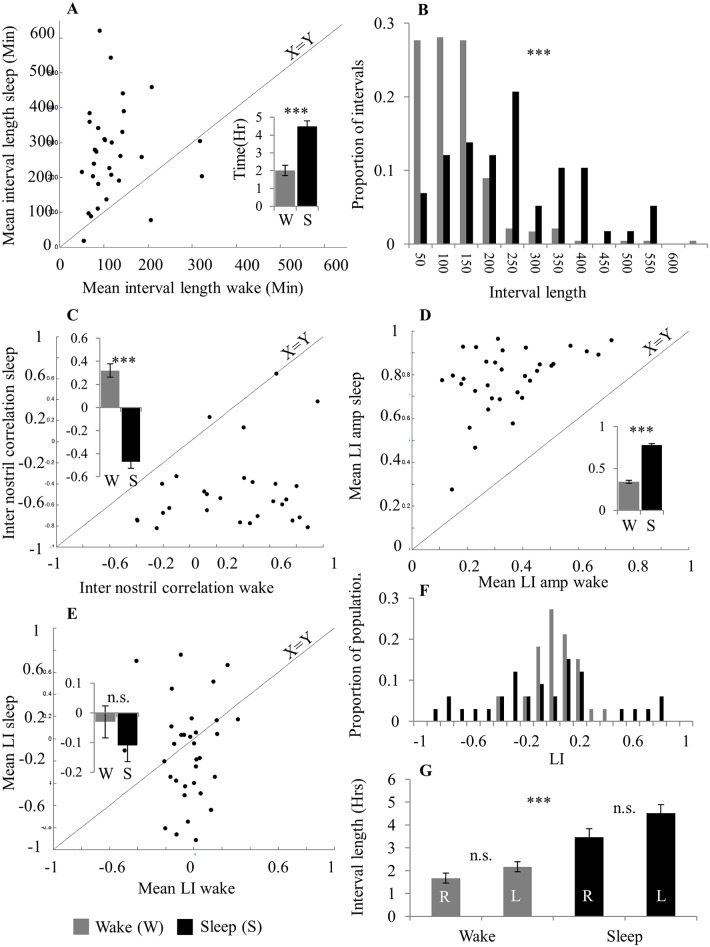
The nasal cycle differed in wake and sleep. (A) Mean interval length. Inset: Mean over population. (B) Distribution of wake and sleep interval lengths across subjects. (C) Inter nostril correlation. Inset: Mean over population. (D) Mean LI amplitude Inset: Mean over population. (E) Mean LI. Inset: Mean over population. (F) Distribution of wake and sleep mean LI across subjects. (G) Right and left interval means during wake and sleep. In scatter plots each dot is a subject and the diagonal line is the unit slope line (X = Y). Error bars are SE.

For each period of sleep and wake we calculated the mean LI, LI amplitude, inter nostril correlation, interval length, right dominant intervals and left dominant intervals. Most nasal cycle parameters were calculated for all 33 subjects but some parameters could not be obtained in some cases (for example, in subjects who did not cycle in sleep). A two-way ANOVA with conditions of arousal state (Wake/Sleep) and parameter (mean LI, LI amplitude, inter nostril correlation, interval length, right dominant intervals and left dominant intervals) revealed significant main effects for arousal state (F(1,32) = 52.25, p < 0.0001) and parameter (F(5,160) = 167.64, p < 0.0001), and an interaction of parameter and arousal state (F(5,160) = 23.6, p < 0.0001). Follow up paired t-tests revealed significant differences between wake and sleep in most cycle characteristics: Cycle length was longer during sleep than wake (mean cycle length sleep = 4.5 ± 1.7 hours, mean cycle length wake = 2.02 ± 1.7 hours, t(30) = 5.73, p < 0.0001, Fig 7A). This difference was evident not only in the averaged cycle length (i.e., one value for wake and one for sleep), but also in the pool of cycle lengths across the population (Kolmogorov–Smirnov test, D = 0.49, p < 10^−10^) (Fig 7B). Moreover, nostrils were anti-correlated during sleep compared to wake (average inter-nostril correlation coefficient in sleep = -0.47 ± 0.33, in wake = 0.32 ± 0.33, paired t-test, t(28) = 8.87, p < 0.0001, Fig 7C). Finally, LI amplitude was higher in sleep than in wake (LI amplitude during Sleep = 0.78 ± 0.1, during Wake = 0.34 ± 0.1, paired t-test, t(32) = 15.63 p < 0.0001, Fig 7D). In contrast to these differences, mean LI was constant across wake and sleep (Mean LI during sleep = -0.11 ± 0.31, during wake = -0.03 ± 0.31, t(32) = 1.0, p = 0.32, Fig 7E). Given that mean LI across the 24 hours was distributed normally around zero (Mean LI = 0.04 ± 0.16, Shapiro Wilk test of normality, SW = 0.9, t(30) = 1.39, p = 0.17, Fig 7F), together these measures imply that the distribution of LI is maintained despite individual differences in sleep duration.

**Fig 8:**
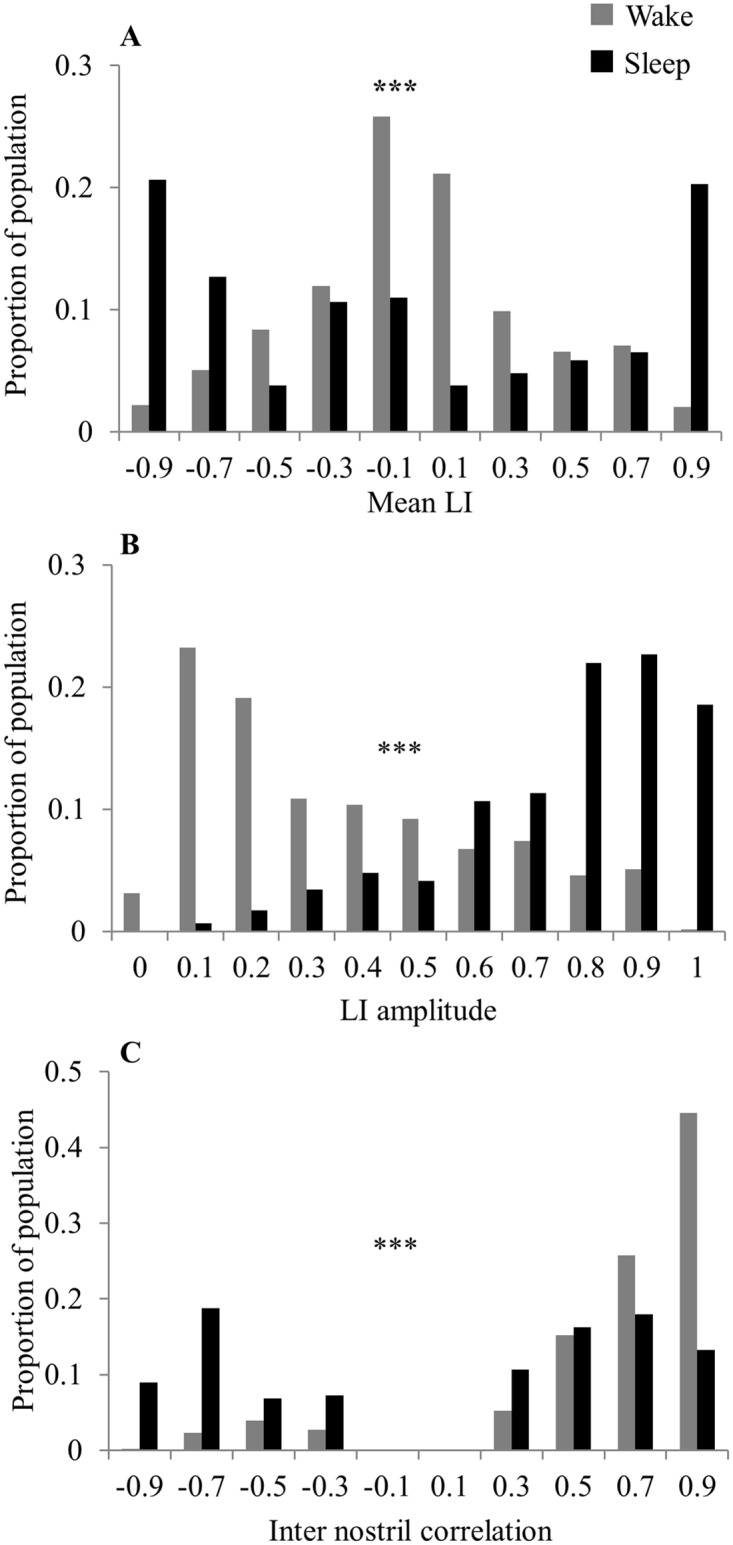
The difference between sleep and wake was evident in 1-hour windows. Distribution of nasal cycle characteristics calculated in 1-hour non-overlapping windows. (A) Mean Laterality Index. (B) Amplitude of laterality index. (C) Inter nostril correlation. This figure implies that the results did not reflect an averaging artifact.

All of the above analyses were conducted on the entire periods of either 24 hours, or total wake and total sleep. To verify that the results were not introduced by averaging alone, we calculated the mean LI, LI amplitude and inter-nostril correlation in non-overlapping one hour time bins. We found that the differences between wake and sleep remained intact using this approach (mean LI distributions in wake and sleep, Kolmogorov-Smirnov (KS) test, D = 0.27, p < 10^12^, Fig 8A. LI amplitude distributions in wake and sleep, KS test, D = 0.22, p < 10^−7^, Fig 8B. Inter-nostril correlation in wake and sleep, KS test, D = 0.37, p < 10^−23^, Fig 8C). For inter nostril correlation, analysis of 1-hour bins did change the absolute values of correlation but not the ratio whereby inter nostril correlation during wake was more positive than the inter nostril correlation during sleep. In other words, we observed genuine alterations in the nasal cycle as a function of sleep and wake that were not averaging artifacts.

### Slower breathing was associated with a more powerful cycle

Here we examined a possible link between nasal cycle dominance and high/low respiratory rates. Respiration rate is defined as the number of inhale-exhale cycles per minute. For each subject we calculated the laterality index amplitude (i.e. absolute value of the mean LI) during the 10% of highest and lowest respiration rate points.

**Fig 9:**
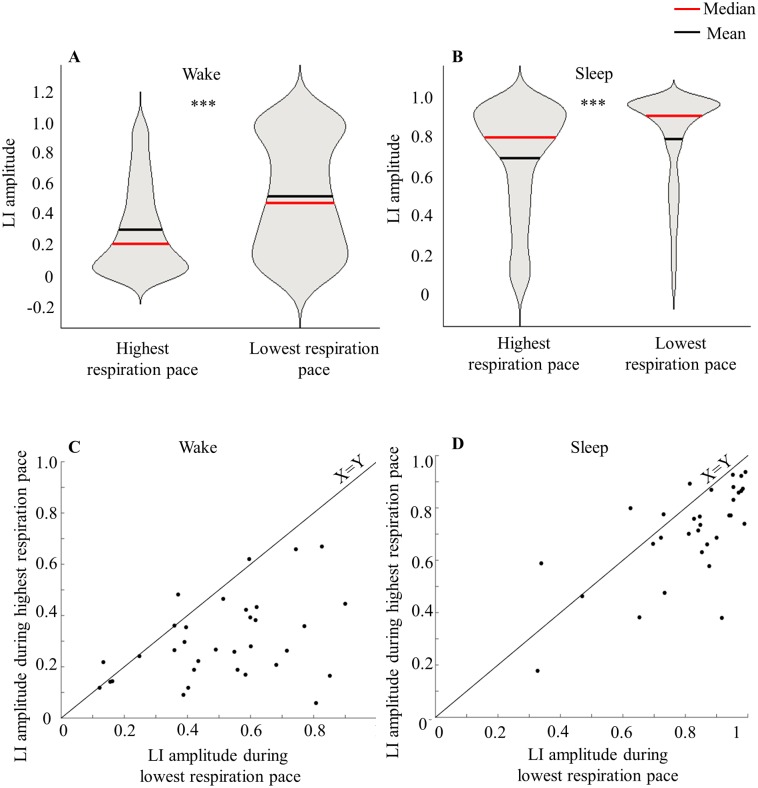
The nasal cycle is linked to overall respiratory frequency. (A) LI amplitude as reflected during highest and lowest respiration pace at wake. (B) LI amplitude as reflected during highest and lowest respiration pace at sleep. Note: We chose violin presentation for A and B panels as it demonstrates not only population mean but also the median and distribution of LI during different respiration conditions. Note that although mean and median values are correct, violin presentation deforms the distribution to be greater than 1, a case clearly not feasible for LI. (C) Comparison of mean LI amplitude during lowest vs highest respiration pace for each subject during wake. (D) Comparison of mean LI amplitude during lowest vs highest respiration pace for each subject during sleep.

Overall respiratory rate varies throughout the day, and across sleep and wake [97]. In the current cohort, mean respiratory rate was 21 ± 0.55 breathes/min during wake, and 18.77 ± 0.55 breathes/min during sleep (paired t-test t(32) = 16.47 p < 0.0001). We found that mean LI amplitude was significantly different between low and high respiratory rate and this significant difference was observed in both wake and sleep (mean LI amplitude during low respiratory rate (wake) = 0.51 ± 0.21, during high (wake) = 0.30 ± 0.16, paired t-test, t(32) = 5.8 p < 10^−5^, Fig 9A. Mean LI amplitude during low respiratory rate (sleep) = 0.82 ± 0.18, during high (sleep) = 0.7 ± 0.18, paired t-test, t(31) = 4.24 p < 0.001, Fig 9B). This link between nasal cycle and respiratory rate was not only evident at the population level but also at the individual subject level (Fig 9C and 9D). The link between nasal cycle and respiratory rate was manifested in LI amplitude but not LI mean. In other words, during slow respiration dominance is extreme, yet during rapid respiration flow tends to balance across nostrils. Notably, this phenomenon was also evident in wake and sleep separately and therefore was not merely a reflection of slower respiratory frequency in sleep.

### Nasal dominance was related to body posture

**Fig 10:**
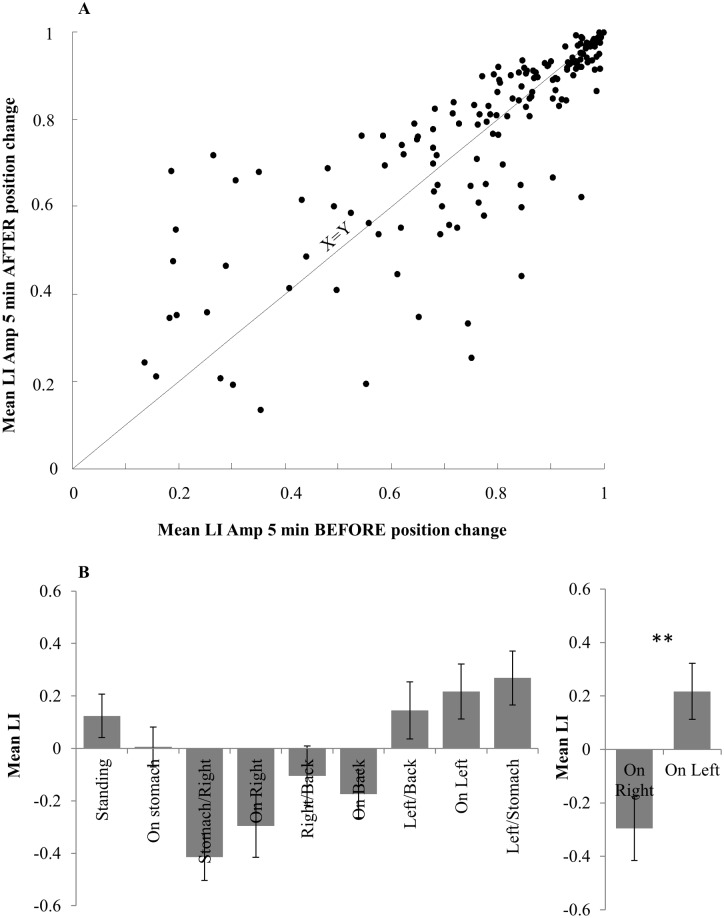
Relation between nasal cycle and body posture. (A) Mean LI amplitude directly before and after position change. Data shown for all types of position change (To right, to left, to stomach, to back etc) pooled over 23 subjects. No significant difference is observed between directly before and after position change, indicating that position change alone does not produce an artifact in LI amplitude during sleep. (B) Correlation between Laterality index and body posture. Left: mean LI for each measured body position. Right: Comparison between ‘on right’ and ‘on left’ position LI means.

A possible concern for our measure of the nasal cycle during sleep is a mechanical artifact reflecting blockage of one cannula during a recumbent position. To address this we extracted data from the position logger. When averaging LI amplitude over 5 minutes time windows before versus after all position changes, we found no significant difference between the two (t(165) = 0.4, p = 0.68, n.s. Fig 10A). This indicates that position change alone did not trigger an artifact of nostril dominance. In turn, consistent with previous reports (Hasegawa, 1982; Eccles 2000; Haight & Cole 1989, 1986), we observed an overall contra lateral relation between body posture and nostril dominance. We averaged LI for each position and each subject. A one way ANOVA on the mean LI in each of 9 position categories (standing, and the formally described 8 lying positions) uncovered a significant position effect (F(8,168) = 7.01, p < 0.0001, Fig 10B). Post-hoc Tukey testing comparing the differences between all positions revealed a significant difference between ‘on right’ and ‘on left’ (mean LI during left recumbent position = 0.21 ± 0.37 mean LI during right recumbent position = -0.29 ± 0.37, p < 0.002, Fig 10B). There were no other significant differences (all p > 0.05). In sum, body position was correlated with nostril dominance in a contra-lateral manner: lying on the left side shifts respiration toward right dominance and vice versa.

### Cyclic flow was evident in each nostril alone

**Fig 11:**
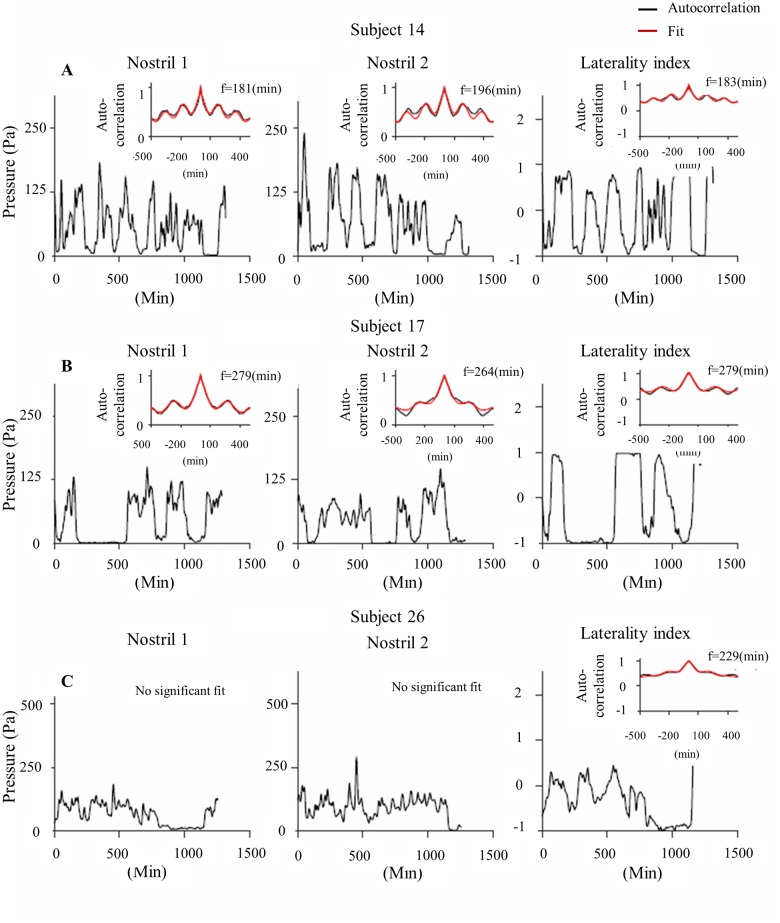
Autocorrelation revealing oscillations in single nostrils: Examples from 3 individual subjects. Twenty-four hour respiration envelopes of right nostril (left) and left nostril (middle) aligned with the corresponding laterality index calculation (Right). Inset: Autocorrelation results for each graph (black) with the fitted model (red–see Methods for fit details). (A) Subject 14 demonstrating similar significant 1/f fluctuation frequency in both right and left nostrils as well as in Laterality Index. This fits with the classical model of both nostrils fluctuating in similar out of phase rhythm resulting in LI with the same rhythm. (B) Subject 17 is also showing similar 1/f fluctuation frequency in right and left nostrils but here LI frequency is obviously contributed from nostril 1 as they are identical. (C) Subject 26 is demonstrating a rather noisy airflow in both right and left nostrils with no distinct oscillation therefore no significant value is detected by the model. Yet, a significant oscillation is detected in the laterality index vector indicating of significant reciprocal changes between the nostrils.

The classical view of the nasal cycle was established through descriptive observations generating the notion that one frequency will dominate the airflow fluctuations in both nostrils and thus the nostril dominance switch will also fluctuate at this same frequency [98,99]. However, a prior study anecdotally observed some independent airflow oscillations in each nostril, orthogonal to the dominance cycle [90]. Given that this observation was made during an 8-hour study with 1-hour resolution, we further investigated this here during 24 hours at 1-minute resolution. We used autocorrelation analysis for each nostril alone as described in the methods section (see examples in Fig 11A–11C). We fit each autocorrelation function with a decaying cosine function to find the dominant frequency for each nostril. There may be several prominent frequencies in each nostril (overlapping in time or active in separate time domains), but for simplicity, we chose here to focus on the most prominent frequency that explains most of the amplitude of the cross-correlation. We observed a good fit (see Methods) for 63% (21/33) of all subjects. In the majority of the fitted subjects (76%, 16/21)—only one of the nostrils was oscillatory, while in 24% (5/21) significant oscillations were observed in both nostrils. In some cases the two nostrils oscillated at a similar frequency, but the laterality index frequency was clearly dominated by one of the nostrils (Fig 11A and 11B). In some subjects neither nostril exhibited a dominant frequency, yet the laterality index revealed an oscillation in switch from side to side (Example in Fig 11C). In other subjects we observed a significant oscillation only in one nostril, with no prominent characteristic frequency for the other nostril.

**Fig 12:**
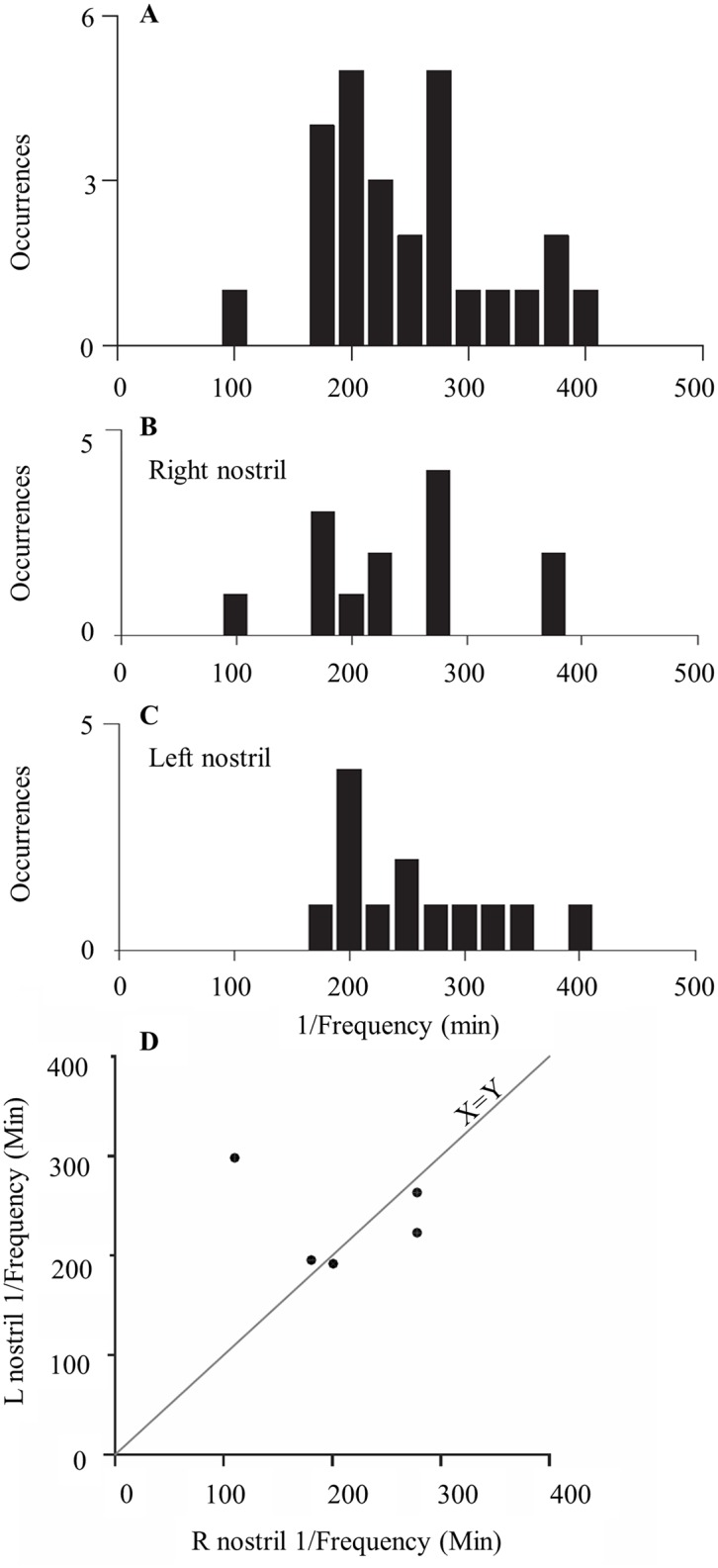
Distribution of single nostril frequencies. (A) Distribution of all frequencies over all significantly oscillatory nostrils, (right and left nostrils pooled together, see Methods for significance threshold). (B) Right nostril frequencies. (C) Left nostril frequencies. (D) Dominant frequency of left nostril vs dominant frequency of right nostril for five subjects in which both nostrils passed significance level.

The distribution of fluctuation frequencies of right and left nostrils pooled together are presented in Fig 12A. The mean fluctuation did not differ between left and right nostrils (mean right nostril fluctuation = 238±60 minutes (Fig 12B), mean left nostril fluctuation = 249.1±60 minutes (Fig 12C), t(25) = 0.61,p = 0.54 n.s.). Fig 12D shows the relations between the left and right dominant frequencies for the five subjects in which we identified a prominent oscillation in both nostrils. We further looked at the correlation between the frequencies in the 5 subjects where both of the nostrils were significant (Fig 12D): in 4/5 cases there was a high positive correlation between the two frequencies (see 4 examples near the identity line). Together, these analyses suggest that airflow may oscillate at an independent frequency in each nostril.

## Discussion

In order to effectively characterize the nasal cycle we developed a small portable device that enables long-term recordings. This device is affordable, easy to build and easy to use. We then derived several numerical measures one can use to characterize the nasal cycle, and obtained these measures from 33 healthy subjects, providing for the largest published cohort of nasal cycle recordings that we are aware of. This effort yielded several results, in part verifying previous observations, and in part providing novel insight into the phenomenon of the nasal cycle.

In agreement with previous efforts, we observed significant variability in cycle across subjects, with average cycle duration of 2.02 ± 1.7 hours in wake and 4.5 ± 1.7 hours in sleep. The variability across subjects may reflect sensitivity of the nasal cycle to behavioral, hormonal and physiological state. The nasal cycle reflects physiological factors such as axillary sweat production [100], pupil size [101], rhinal activity [101], rhythms of the neuroendocrine, cardiovascular and insulin systems [102,103], and most importantly from our perspective, with brain activity [41]. Indeed, we found a strong link between the nasal cycle and level of overall arousal as evidenced in the sleep wake cycle. Consistent with recent studies [104,105], we found that nasal cycle alterations were more frequent in wake, but the amplitude of difference was greater in sleep. Moreover, consistent with previous studies, we found that the nasal cycle was influenced by body posture such that lying on one side was associated with greater flow in the contralateral nostril [28,29,93]. Finally, we found a small but significant population bias towards left nostril dominance in this cohort of right-handed healthy young individuals. That said, a potential weakness of this study is that we did not subject participants to a nasal exam by physician. Thus, it remains possible that an unidentified group abnormality such as undiagnosed deviated septum, etc., may underlie in part the observed bias towards left nostril dominance.

Whereas the above results echoed several previous findings and in this served to validate our device and method, this effort also yielded novel results as well. First, the periodicity of the nasal cycle was related to overall respiratory frequency, with slower respiration associated with a more powerful nasal cycle. Analysis in wake alone reveled that this was not merely a reflection of altered respiration in sleep and wake.

A potential mechanistic underpinning for this relationship is a common neuronal substrate in the brainstem reticular formation that is linked with vasomotor regions [106], modulated with arousal [107,108], and involved in modulation of the nasal cycle [109,110] and respiration in general [111,112]. A second mechanism potentially related to this observation is mediation via changes in levels of expired CO_2_. The nasal cycle is associated with asymmetric end-tidal CO_2_ [113]. Slower breathing raises CO_2_ concentrations overall, and elevated CO_2_ reduces nasal congestion [114]. However, given that nasal vasomotor responses are typically greater on the congested or low airflow side of the nose [115], levels of expired CO_2_ could in fact act to weaken rather than increase the power of the cycle during slow respiration. Thus, we conclude that our observation of a more powerful cycle during slow respiration was robust, but we have no strong model explaining this result.

The second novel finding obtained in this study was that each nostril alone may have an underlying oscillation in flow that is unrelated to the nasal cycle. This finding calls for careful follow-up in order to identify the driving mechanisms of this phenomenon, and its indicative power regarding general physiological and neurological function if any. While the analysis we used is rather conservative it still enabled an exact evaluation of the nostril fluctuation frequency for many subjects. This may serve as the basis for future studies that may use this subject-personal-fingerprint to characterize individual processes related to the nasal cycle in both health and disease.

In conclusion, we provide mechanical instructions for construction of a simple and rugged logging device, and analysis schemes that allow deduction of several meaningful nasal cycle parameters. Given the indicative value of the nasal cycle for overall arousal and neural asymmetry, we hope this device will allow for extensive further characterization of this marker in both health and disease.
